# Prediction of Alzheimer's disease using an AI driven screening platform: interim results on feasibility, usability and acceptability of home‐based assessments in the PREDICTOM study

**DOI:** 10.1002/alz70858_101010

**Published:** 2025-12-24

**Authors:** Anna‐Katharine Brem, Zunera Khan, Ellie Pickering, Martha Therese Gjestsen, Johanna Mitterreiter, Nicholas J. Ashton, Martin Pszeida, Bin Huang, Sigurd Brandt, Richard Layton, Sarah Campill, Dianne Gove, Ingelin Testad, Spiros Nikolopoulos, Anne Corbett, Dag Aarsland, PREDICTOM

**Affiliations:** ^1^ King's College London, London, United Kingdom; ^2^ Institute of Psychiatry, Psychology and Neuroscience, King's College London, London, United Kingdom; ^3^ University of Exeter, Exeter, United Kingdom; ^4^ Centre for Age‐Related Medicine – SESAM, Stavanger University Hospital, Stavanger, Norway; ^5^ Siemens Healthineers AG, Forchheim, Germany; ^6^ Banner Sun Health Research Institute, Sun City, AZ, USA; ^7^ Banner Alzheimer's Institute and University of Arizona, Phoenix, AZ, USA; ^8^ JOANNEUM RESEARCH Forschungsgesellschaft mbH, Graz, Styria, Austria; ^9^ BrainCheck Inc., Austin, TX, USA; ^10^ GN brainworks, Ballerup, Denmark; ^11^ Muhdo, Ipswich, United Kingdom; ^12^ Alzheimer Europe, Luxembourg, Senningerberg, Luxembourg; ^13^ Alzheimer Europe, Senningerberg, Luxembourg, Luxembourg; ^14^ Centre for Research & Technology Hellas, Thessaloniki, Greece; ^15^ College of Medicine and Health, University of Exeter, Exeter, United Kingdom; ^16^ Centre for Age‐Related Medicine, Stavanger University Hospital, Stavanger, Stavanger, Norway; ^17^ Kings College London, London, United Kingdom

## Abstract

**Background:**

Early prediction of Alzheimer's disease (AD) is a critical healthcare challenge. The PREDICTOM study leverages technological advances in neurophysiological and digital biomarkers to develop an AI‐driven platform enabling home‐based early diagnostic assessment. Here, we assess the feasibility, usability, and acceptance of collecting digital and physiological biomarkers in the home‐setting.

**Method:**

In this multicentre study *N* = 4000 individuals over the age of 50 with increased risk of developing AD are recruited across 7 European sites. Level 1 includes a home‐based assessment including digital (cognition, hearing, eye‐tracking, questionnaires) and physiological (finger‐prick blood, saliva) biomarkers, which is followed by more in‐depth in‐clinic measures in Levels 2 and 3. Here, we will assess (1) feasibility (tasks and questionnaires completed, biomarkers provided, drop‐outs), (2) usability (feedback on usability of sampling kits for finger‐prick blood and saliva, number of times help is needed by contacting clinical sites or helpdesk), and (3) acceptability (evaluation of the study protocol in terms of time investment and complexity and acceptability of our feedback materials). Furthermore, we will assess differences between clinical sites that administer tests remotely as opposed to a quasi‐remote setup taking place within a clinical setting and assess the impact of cognitive function and on these measures across people with and without AD pathology using MANCOVA (covariates: age, education, sex).

**Result:**

The interim analysis of Level 1 data will include *N*»1000 across the spectrum from high to low risk of AD.

**Conclusion:**

The PREDICTOM study will offer pioneering insights into at‐home AD diagnostic measures, with initial results providing preliminary evidence of feasibility, usability and acceptability. At AAIC, a full readout of the data from the first *N*»1000 participants from PREDICTOM will be presented.

**Acknowledgment**

This Project Is Supported By The Innovative Health Initiative Joint Undertaking (IHIJU) Under Grant Agreement No101132356. The JU Receives Support From The European Union's Horizon Europe Research And Innovation Programme. This Work Was Funded By UK Research And Innovation (UKRI) Under The UK Government's Horizon Europe Funding Guarantee [UKRI Reference Number:10083181]. In Switzerland The University Of Geneva Is Funded For PREDICTOM By The Swiss State Secretariat For Education Research And Innovation (SERI‐ Ref‐1131 52304).

